# A 5-Fluorouracil-Induced Hyperammonemic Encephalopathy Challenged with Capecitabine

**DOI:** 10.1155/2020/4216752

**Published:** 2020-01-31

**Authors:** Michael Chahin, Nithya Krishnan, Hardik Chhatrala, Marwan Shaikh

**Affiliations:** ^1^University of Florida College of Medicine–Jacksonville, Department of Medicine, Division of Internal Medicine, USA; ^2^University of Florida College of Medicine–Jacksonville, Department of Medicine, Division of Hematology and Oncology, USA

## Abstract

Cancer patients presenting with altered mental status demand a broad differential with early recognition of the etiology. Failure to do so is associated with increased morbidity and mortality. Causes that must be considered include organ involvement of the cancer, electrolytes abnormalities, and even chemotherapeutic agents. A 32-year-old female patient had been recently started on FOLFOX for metastatic colon cancer. Her initial treatments were uneventful, but she later developed encephalopathy during day three of cycle five. During her evaluation, she was found to have hyperammonemia (84 mcmol/L), without hepatic failure, that resolved with stopping chemotherapy and supportive care. After a trial of home infusion fluorouracil, she developed hyperammonemic encephalopathy again. During both admissions, her symptoms resolved with IV hydration and cessation of chemotherapy. She was then successfully challenged with capecitabine (1000 mg/m^2^ daily), and additional hydration, and continued chemotherapy without recurrence of symptoms. Hyperammonemia is associated with fluorouracil though the mechanism is unclear. Suspected etiologies include either elevated levels of the drug due to slower metabolism or accumulation of certain metabolites. Additionally, risk factors such urease-producing bacterial infections, dehydration, and increased catabolism are thought to increase the risk for hyperammonemia. This case demonstrates the need for greater awareness of fluorouracil as a cause of hyperammonemic encephalopathy. Knowledge of this may allow for earlier recognition and reduced unnecessary testing.

## 1. Introduction

Confusion in the cancer patient can present a diagnostic dilemma. It is often multifactorial, possibly due to the delirium from the malignancy itself, electrolyte abnormalities, organ involvement, and even the cancer treatment. Certain chemotherapy drugs, such as cytarabine and methotrexate, are associated with delirium. Early diagnosis is crucial as delirium in the cancer patient conveys increased morbidity and mortality [1]. Factors that increase the risk for the development of delirium include age > 70 years, cognitive impairment, and end-stage organ dysfunction [2]. One must keep in mind that these patients often develop hypoactive delirium, which could delay diagnosis if not recognized [1, 3]. We present a patient who developed hyperammonemic encephalopathy following treatment with 5-fluorouracil (5-FU) for metastatic colon cancer.

## 2. Case Presentation

A 32-year-old female with a history of iron deficiency anemia presented with bright red blood per rectum and worsening anemia that lead to the diagnosis of an invasive rectosigmoid colonic adenocarcinoma. The tumor was microsatellite stable, KRAs mutated, and BRAF wild type. A positron emission tomography scan revealed increased uptake in the rectosigmoid lesion, retroperitoneal lymph nodes, and numerous liver lesions. Brain magnetic resonance imaging did not show brain metastasis. Her Eastern Cooperative Oncology Group (ECOG) Performance Status was zero.

She was started on FOLFOX and bevacizumab chemotherapy. Her regimen was folinic acid 400 mg/m^2^ once on day one, 5-FU 400 mg/m^2^ bolus on day one followed by 2400 mg/m^2^ continuous infusion over 46 hours, oxaliplatin 85 mg/m^2^ once on day one, and bevacizumab 5 mg/kg once on day one. She tolerated cycles 1-4 with mild nausea and vomiting. During the third day of cycle five, she became acutely altered and developed nausea and vomiting beyond what she had with the initial two days of chemotherapy. The 46-hour infusion of 5-FU has been completed the night before, and she had presented to the infusion center that morning for pump removal. An emergency department evaluation was advised due marked lethargy. She was oriented to person and place but not to time or situation. Her gait was ataxic, she a mild tremor, and her speech was minimally comprehensible. Her Glasgow Coma Score (GCS) was 12. Her spouse endorsed that she felt weak and had some vomiting the night before. No recent constipation. CT head was unremarkable (Table 1).

She was given intravenous fluids, observed overnight, and discharged home after symptom resolution. Her lactic acidosis and acute kidney injury resolved. Empiric broad spectrum antibiotics had been given but were stopped due to negative infectious workup and rapid improvement with hydration. She received lactulose and her chemotherapy was held while inpatient. She did not recall much from her presentation.

For cycle six of chemotherapy she was started on 5-FU alone for a home infusion over 46 hours, folinic acid, and bevacizumab therapy. On day one of treatment, shortly after starting the 5-FU infusion, she again developed altered mental status. Her physical exam was significant for asterixis, nonverbal status, and not following commands. She was not ambulating. Her GCS was eight this time. She was treated with fluids until symptom resolution and was discharged. Lactulose had been resumed while inpatient, and she was to continue it at discharge (Table 2).

Having developed hyperammonemic encephalopathy in the previous two chemotherapy regimens, she was then switched to capecitabine 1000 mg/m^2^ and oxaliplatin (CAPEOX) and bevacizumab. Her weight had remained stable since starting chemotherapy, but she had appeared dehydrated during clinic visits. She was encouraged to take in more fluids and was given IV fluids during chemotherapy sessions. She was advised to cease using lactulose since there was no overt hepatic failure. She had no recurrence of hyperammonemia symptoms while on CAPEOX, for a total of eight cycles. A dihydropyrimidine dehydrogenase (DPD) level had been ordered during the previous admission and was normal. Based on these findings, her hyperammonemia was attributed to 5-FU.

## 3. Discussion

Colon cancer is the third most common cause of cancer-related death in the United States [4]. The mainstays of chemotherapy are folinic acid, 5-fluorouracil, and oxaliplatin (FOLFOX), and folinic acid, 5-FU, and irinotecan (FOLFIRI) [5]. Capecitabine and oxaliplatin (CAPOX) can be used as an oral alternative to FOLFOX [5, 6]. This patient tolerated capecitabine, without recurrence of her hyperammonemia.

The mechanism of fluorouracil-induced hyperammonemic encephalopathy is unclear but likely multifactorial. Deficiency in DPD, the primary enzyme in the metabolism of 5-FU, may play a role though this patient's DPD activity was normal [7]. Another possible etiology is inhibition of the Krebs cycle by fluoroacetate or fluorocitrate, metabolites of 5-FU, leading to excess urea and the subsequent accumulation of ammonia [7, 8].

It has been proposed as well that 5-FU therapy alone does not generally induce hyperammonemia. Rather, weight loss, dehydration, constipation, bacterial infection, and renal impairment are predisposing factors. Chronic constipation leading to increased bacterial urease and amino acid oxidase activity and increased catabolism in chronically anorexic patients also contribute to a hyperammonemic state [9]. Urinary tract infection due to urease-producing bacteria is associated with hyperammonemia, independently of 5-FU [10]. Renal impairment is thought to increase levels of 5-FU and 5-fluoro-beta-alanine (FBAL), another metabolite of 5-FU [9, 11]. Direct neurotoxicity from these agents is possible based on a study conducted in cats that revealed, similar, neuropathologic changes in subjects with intraventricular administration of FBAL and 5-FU given orally [12].

Hyperammonemia, in general, is most commonly due to liver damage but there are many other causes that must be considered. Some causes, in addition to urinary tract infection, are due to urease-producing bacteria, excessive amino acid load from gastrointestinal hemorrhage, anticonvulsants, such as topiramate, and urea cycle defects [10]. Multiple chemotherapy agents, in addition to 5-FU, have been implicated in hyperammonemic encephalopathy [13]. Though these have not been specifically associated with fluorouracil-induced hyperammonemic encephalopathy, they likely predispose patients to developing it. A case series demonstrated hyperammonemic encephalopathy in 5-FU and oral fluoropyrimidine agents. Most of the patients had risk factors in common such as dehydration and sarcopenia [14].

A rechallenge protocol has been suggested in the context of hyperammonemic encephalopathy after receiving 5-FU, oxaliplatin, irinocetan, and folinic acid (FOLFIRINOX) [15]. The management of the patient presented above was focused on mitigating her primary risk factor for hyperammonemia, which was dehydration. It is likely that the acute kidney injury in this patient was a result of dehydration but could still have been a risk factor for hyperammonemia.

## 4. Conclusion

Given the prevalence of colon cancer, the primary care, hospitalist, and emergency physician should be wary of 5-FU causing hyperammonemia. This patient was treated with lactulose despite not having hepatic damage. Though one should have a broad differential, in a cancer patient, presenting with confusion recognition of this adverse effect of 5-FU may reduce unnecessary testing. Additionally, this case demonstrates that patients with 5-FU-induced hyperammonemic encephalopathy may be challenged with capecitabine though the risk of encephalopathy remains. Treatment should be focused on reducing risk factors for hyperammonemia.

## Figures and Tables

**Table 1:** 

	Reference range	Two days before admission	Day of admission
Sodium	135-145 mmol/L	142	144
Potassium	3.3-4.6 mmol/L	4.0	4.9
Chloride	101-110 mmol/L	106	108
Carbon dioxide	21-29 mmol/L	25	13
Urea nitrogen	6-22 mg/dL	9	17
Creatinine	0.51-0.95 mg/dL	0.65	0.96
Glucose	71-99 mg/dL	96	73
Calcium	8.6-10.0 mg/dL	9.4	9.4
Anion gap	4-16 mmol/L	11	23
Total protein	6.5-8.3 g/dL	7.3	7.7
Albumin	3.8-4.9 g/dL	4.3	4.6
AST	14-33 IU/L	17	40
ALT	10-42 IU/L	14	48
Direct bilirubin	0.0-0.2 mg/dL		0.3
Indirect bilirubin	<0.8 mg/dL		1.4
Total bilirubin	0.2-1.0 mg/dL	0.4	1.7
Alkaline phosphatase	35-104 IU/L	66	82
Ammonia	11-35 mcmol/L		84
Lactic acid	0.7-2.7 mmol/L		10.4

**Table 2:** 

	Reference range	Day of second admission
Sodium	135-145 mmol/L	139
Potassium	3.3-4.6 mmol/L	4.5
Chloride	101-110 mmol/L	101
Carbon dioxide	21-29 mmol/L	15
Urea nitrogen	6-22 mg/dL	18
Creatinine	0.51-0.95 mg/dL	1.13
Glucose	71-99 mg/dL	94
Calcium	8.6-10.0 mg/dL	10.6
Anion gap	4-16 mmol/L	23
Total protein	6.5-8.3 g/dL	9.0
Albumin	3.8-4.9 g/dL	5.1
AST	14-33 IU/L	24
ALT	10-42 IU/L	27
Direct bilirubin	0.0-0.2 mg/dL	0.3
Indirect bilirubin	<0.8 mg/dL	1.1
Total bilirubin	0.2-1.0 mg/dL	1.4
Alkaline phosphatase	35-104 IU/L	89
Ammonia	11-35 mcmol/L	290
Lactic acid	0.7-2.7 mmol/L	8.2
